# Platelet parameters may not be related to new-onset atrial fibrillation after coronary bypass surgery

**DOI:** 10.1590/1806-9282.20240729

**Published:** 2024-09-16

**Authors:** Cengiz Beyan

**Affiliations:** 1 Ufuk University, Faculty of Medicine – Ankara, Turkey.

Dear Editor,

I read with great interest the retrospective study of Demirel et al., which investigated the existence of a seasonal relationship between platelet count and mean platelet volume (MPV) values and the risk of postoperative atrial fibrillation in patients who underwent coronary artery bypass graft (CABG) surgery in spring and autumn^1^. They found preoperative MPV and platelet mass index (PMI) values to be significantly higher in patients who operated in the autumn months compared to the spring months and observed a significant relationship between PMI and postoperative atrial fibrillation. I would like to emphasize the existence of some factors that may have negatively affected the platelet parameters in the results of this study.

Although the authors suggested that platelet parameters are related to the pathogenesis of inflammation, MPV measurement methodology has not been standardized to date, and therefore it is not recommended to use MPV values for diagnosis or prognosis, especially in acquired diseases^2^. The main factors that directly affect MPV measurement standardization are the time from blood collection to MPV measurement, the anticoagulant used in MPV measurement, and the devices used in MPV measurement^3–8^. Ethylenediaminetetraacetic acid (EDTA) is the most commonly used anticoagulant in complete blood counts, and contact with EDTA in the blood tube causes platelets to rapidly swell, increase their diameter, and develop into podocytes^3^. This diameter increase in platelets can increase up to 30% in the first 5 min and 40–45% in the first 2 h^3^. In studies using EDTA, an MPV increase of 2–50% has been reported^3,5^. The use of other anticoagulants also causes an increase in MPV^4,9^. Lancé et al. aimed to standardize the time from blood collection to MPV measurement and found the optimal MPV measurement time to be 60 and 120 min after blood collection, respectively, based on the use of sodium citrate and dipotassium EDTA as anticoagulants^4,9^. Differences in blood analyzers used in measurement also cause deviations in MPV values of up to 40%^5–8^. Since PMI is a parameter obtained by calculation as a result of multiplying MPV and platelet count, any standardization problem that affects MPV values directly affects PMI values. The fact that no methodology for MPV measurement was defined in the study of Demirel et al. significantly negatively affects the reliability of the measured MPV and calculated PMI results. Likewise, the fact that the study was conducted retrospectively made it impossible to rule out preanalytical and analytical errors, and this is an unacceptable situation for MPV studies^10^.

Contrary to the authors’ claim, another important point that should be emphasized is that MPV values are related to platelet production, not platelet function. The gold-standard measurement of platelet function is performed by light transmission platelet aggregometry performed in platelet-rich plasma^11^, and in studies using this technique, no correlation was found between all platelet parameters, including MPV and PMI, and platelet aggregation responses to aggregation-stimulating agents^12,13^.

As a result, preoperative MPV and PMI values may not be associated with seasonal changes and postoperative atrial fibrillation in patients undergoing CABG surgery.
